# Dimensionality assessment in ordinal data: a comparison between parallel analysis and exploratory graph analysis

**DOI:** 10.3389/fpsyg.2024.1359111

**Published:** 2024-05-06

**Authors:** Angelos Markos, Nikolaos Tsigilis

**Affiliations:** ^1^Department of Primary Education, Democritus University of Thrace, Alexandroupolis, Greece; ^2^Department of Journalism and Mass Media, Aristotle University of Thessaloniki, Thessaloniki, Greece

**Keywords:** factor analysis, factor retention, polychoric correlation, scale validation, simulation study

## Abstract

In the social sciences, accurately identifying the dimensionality of measurement scales is crucial for understanding latent constructs such as anxiety, happiness, and self-efficacy. This study presents a rigorous comparison between Parallel Analysis (PA) and Exploratory Graph Analysis (EGA) for assessing the dimensionality of scales, particularly focusing on ordinal data. Through an extensive simulation study, we evaluated the effectiveness of these methods under various conditions, including varying sample size, number of factors and their association, patterns of loading magnitudes, and symmetrical or skewed item distributions with assumed underlying normality or non-normality. Results show that the performance of each method varies across different scenarios, depending on the context. EGA consistently outperforms PA in correctly identifying the number of factors, particularly in complex scenarios characterized by more than a single factor, high inter-factor correlations and low to medium primary loadings. However, for datasets with simpler and stronger factor structures, specifically those with a single factor, high primary loadings, low cross-loadings, and low to moderate interfactor correlations, PA is suggested as the method of choice. Skewed item distributions with assumed underlying normality or non-normality were found to noticeably impact the performance of both methods, particularly in complex scenarios. The results provide valuable insights for researchers utilizing these methods in scale development and validation, ensuring that measurement instruments accurately reflect theoretical constructs.

## 1 Introduction

In the social sciences, particularly within psychological and educational research, measurement instruments, or scales, play a pivotal role in assessing latent constructs such as anxiety, happiness, and self-efficacy, which cannot be directly observed (Hayton et al., 2004; Costello and Osborne, 2005; Cho et al., 2009). These scales are essential for quantifying and analyzing various facets of human behavior. A critical aspect of developing and utilizing a scale is the understanding of its dimensionality, which refers to the number and types of latent factors that impact the observed variables. Accurate determination of a scale’s dimensionality is crucial, as it ensures the instrument’s alignment with its conceptual framework and theoretical foundations (Hayton et al., 2004; Costello and Osborne, 2005; Cho et al., 2009).

Exploratory factor analysis is a widely used technique in scale development. Its employment helps researchers to identify the number of underlying factors influencing observed variables (Costello and Osborne, 2005). The literature suggests several criteria for this purpose, including eigenvalues greater than one (Kaiser, 1960), the scree test (Cattell, 1966), the minimum average partial (MAP) correlation procedure (Velicer, 1976), parallel analysis (PA) (Horn, 1965; Hayton et al., 2004), revised parallel analysis (Green et al., 2012) and, more recently, exploratory graph analysis (EGA) (Golino and Epskamp, 2017). PA is often cited as one of the most reliable methods for assessing dimensionality (Hayton et al., 2004; Costello and Osborne, 2005; Goretzko et al., 2021), consistently outperforming other criteria in analyzing continuous (Crawford et al., 2010; Auerswald and Moshagen, 2019), dichotomous, and ordinal data (Tran and Formann, 2009; Yang and Xia, 2015). EGA, introduced by Golino and Epskamp (2017) and further investigated in subsequent studies (Golino et al., 2020; Cosemans et al., 2022), employs network analysis techniques to visualize and determine data set dimensionality. It has shown effectiveness for continuous and dichotomous data, suggesting a novel approach to factor analysis that could supplement or, in some cases, replace traditional methods.

Despite these advancements, a significant gap remains in the empirical examination of PA and EGA, especially concerning their application to ordinal data—typical in Likert-scale assessments in education and psychology. The main challenge involves accurately identifying the dimensionality of scales utilizing ordinal responses without assuming an underlying normally distributed latent variable. Given these considerations, our study aims to thoroughly evaluate the effectiveness of PA and EGA in retaining the dimensionality of both unidimensional and multidimensional scales, with a specific emphasis on simulated ordinal data characterized by diverse underlying distributions. The study aims to provide practitioners with comprehensive insights, thereby aiding in the selection of the most appropriate dimensionality assessment techniques for their studies.

## 2 Parallel analysis and exploratory graph analysis

Parallel Analysis (PA) is used to determine the appropriate number of factors in a dataset, ensuring that the factors extracted are based on the intrinsic properties of the data rather than on random chance. It generally involves generating random data sets, calculating their eigenvalues, and comparing these to the eigenvalues of the actual data. Factors are retained if their eigenvalue exceeds the threshold set by random eigenvalues. Different implementations of PA exist, some considering the 95th percentile, others the mean of the random eigenvalues as the cutoff. Commonly, PA employs principal components analysis, often found more effective than common factor analysis (Ruscio and Roche, 2012; Auerswald and Moshagen, 2019). The suitability of Pearson vs. polychoric correlations for PA, especially with ordinal data, is debated, with a preference often given to polychoric correlations (Timmerman and Lorenzo-Seva, 2011; Garrido et al., 2013).

Exploratory Graph Analysis (EGA) is a more recent approach that diverges from traditional factor analysis methods and is rooted in network analysis (Golino and Epskamp, 2017). Unlike factor analysis, which presupposes a common cause for observed variables, network analysis takes the perspective that the co-occurrence of human behaviors or psychopathology symptoms arises from mutual influence among these observed variables (Christensen and Golino, 2021). In EGA, variables are conceptualized as nodes within a network, and the associations between variables are depicted as edges in the network structure. Through the clustering of these nodes, EGA identifies latent factors or communities of variables without explicitly assuming latent variables. This usually involves computing partial correlations using a Gaussian Graphical Model with GLASSO regularization and the Extended Bayesian Information Criterion (EBIC) algorithm for model selection (Golino and Epskamp, 2017). Subsequently, a community detection algorithm is applied to group items into clusters, with these clusters being shown to correspond to latent factors (Christensen and Golino, 2021). While the original EGA algorithm had a tendency to identify multiple factors even in cases where data were generated from a unidimensional structure, Golino et al. (2020) addressed this limitation by developing a new EGA algorithm specifically tailored to handle unidimensional structures. It is this refined approach that we employ in the current study.

## 3 Methods

### 3.1 Simulation design

To comprehensively evaluate the performance of both EGA and PA, we devised a simulation design, closely aligned with previous simulation studies (Goretzko and Bühner, 2020, 2022). Our simulations included datasets with sample sizes of 300, 600, or 1,000 observations, reflecting the range typically encountered in real-world research settings. We varied the number of latent factors, generating scenarios with one, two, or four underlying factors, covering unidimensional and multidimensional structures. Primary loadings, chosen from a uniform distribution, ranged from 0.35 to 0.80, categorized as small (0.35–0.50), medium (0.50–0.65), and high (0.65–0.80). Similarly, cross-loadings ranged from 0 to 0.40, categorized as small (0–0.10), medium-sized (0.10–0.20), and high (0.20–0.30). This variation captured different levels of factor saturation and specificity, crucial for evaluating method sensitivity to variable-factor associations. We also considered orthogonal and oblique factor structures, with inter-factor correlations set to 0, 0.3, or 0.6. Additionally, we manipulated the number of manifest variables by including either five or 10 indicators per factor, and varied the number of categories per variable, utilizing either four or five categories, to closely resemble real-world scenarios encountered in psychological and educational measurement and scale development studies.

A population correlation matrix was created for each data set based on the following decomposition:


$$\Sigma =\Lambda \Phi \Lambda^{Τ}+\Psi$$

where Λ represents the factor loading matrix, which contains the loadings of observed variables on the latent factors, Φ denotes the factor correlation matrix, a symmetric matrix with ones on the diagonal and the correlations between different factors on the off-diagonal, Ψ is the covariance matrix among residuals. The diagonal elements of Ψ are the unique variances of each variable and are calculated by subtracting the variance attributed to the factors (the diagonal elements of $\Lambda \Phi \Lambda^{Τ}$ ) from the total variance of each variable.

To simulate ordinal data, the typical approach starts with generating continuous data from a multivariate normal distribution, which then is discretized into a specified number of ordinal categories. In other words, observed ordinal data usually are assumed to reflect normally distributed unobserved continuous variables that have been discretized. In this study, we varied the underlying distribution of the continuous variable to examine its impact on estimation performance, given the common but questionable assumption of underlying normality in polychoric correlations. This assumption is particularly dubious in the social sciences, where variables frequently deviate from normal distribution, potentially biasing the polychoric correlation estimates (Foldnes and Grønneberg, 2020).

Therefore, to investigate how different distributions impact estimation accuracy, we generated continuous data from either a multivariate normal or a multivariate non-normal distribution, given the specific correlation matrix, Σ . To simulate the multivariate normal data, we utilized the mvtnorm package (Genz et al., 2023). In contrast, the multivariate non-normal data were simulated using the independent generator method, as implemented in the R package covsim (Grønneberg et al., 2022), with predetermined univariate skewness and kurtosis set at 2 and 7, respectively. Following the generation of these distributions, we converted the continuous data into ordinal data by discretizing it either using symmetrical item distributions or (positively) skewed item distributions according to the procedure described by Yang and Xia (2015) and used in the simulation study of Goretzko and Bühner (2022). Specifically, for ordinal variables with four categories, symmetric thresholds were set at (−0.67, 0, 0.67) while asymmetric thresholds were (0, 0.43, 0.97). For variables with five categories, symmetric thresholds were (−0.84, −0.25, 0.25, 0.84), and asymmetric thresholds were set at (0.08, 0.25, 0.62, 1.11).

We determined the number of factors for PA and EGA using the polychoric correlation matrix in our estimation process. Initially, a comprehensive simulation was set up, encompassing 3,888 distinct conditions, each replicated 100 times. These conditions were systematically varied, considering factors such as sample size (three levels), number of indicators per factor (two levels), number of latent variables (three levels), levels of inter-factor correlations (three levels), primary loadings (three levels), cross-loadings (three levels), types of generated ordinal data (four levels determined by symmetrical or skewed distributions, with assumed underlying normality or non-normality), and the number of categories per variable (two levels). However, certain combinations were excluded due to irrelevance (e.g., conditions with one latent factor and non-zero inter-factor correlations) or impossibility (e.g., conditions with certain high loadings and correlations that could yield improper solutions for Σ ). Consequently, the final set of conditions analyzed was reduced to 2,616.

### 3.2 Data analysis

All analyses in our study were carried out using R. For PA, we employed the fa.parallel() function from the psych package (Revelle, 2023), setting cor to “poly” and fa to “pc.” For EGA, we used the EGA() function from the EGAnet package (Golino et al., 2020) with its default settings. After conducting the simulation, we utilized a logistic regression model with two-way interactions between the predictor “retention method” (categorized into two levels: EGA or PA) and each of the remaining variables to predict the probability of accurately identifying the number of factors in each scenario (refer to Supplementary Tables S1, S2). In this model, the dependent variable was a binary indicator reflecting the accuracy of each criterion in determining the correct number of factors (1 = correct, 0 = incorrect). The independent variables included dummy variables for sample size, variables per factor, inter-factor correlations, primary and secondary factor loadings, number of categories, types of generated data, and the factor retention method (either EGA or PA), as well as interactions between each method and the remainder of the variables. The odds ratio was calculated for each predictor to measure the effect size. To ensure reproducibility, the dataset, complete simulation code, and results are available at the Open Science Foundation project for this paper.^1^

## 4 Results

Considering all conditions in the study, EGA achieved the highest accuracy, averaging 71.0%, outperforming PA, which had an overall accuracy of 54.5%. In terms of factor retention criteria, both methods were more prone to underfactoring, meaning they often suggested fewer factors than were actually present. EGA exhibited a lower tendency for underfactoring at 27.6%, in contrast to PA’s 44.8%. Overfactoring, or suggesting too many factors, was rare for both methods, occurring in 1.3% of cases for EGA and 0.7% for PA.

Table 1 shows the averaged accuracy of PA and EGA across a wide range of simulated conditions. Both methods showed high accuracy (often 100%) in identifying the correct number of latent factors in scenarios with well-defined factor structures (high loadings, low or no cross-loadings), particularly at larger sample sizes. Accuracy dropped significantly for both methods in scenarios with low loadings and high inter-factor correlations. This drop is more pronounced for PA, indicating EGA’s relative robustness in these conditions. Both methods experienced a notable decrease in accuracy in scenarios with non-normal or skewed data distributions.

**Table 1:** Mean accuracy (%) of Parallel Analysis and Exploratory Graph Analysis across selected conditions: influence of distribution type, number of factors, inter-factor correlations, variables per factor, factor loadings, cross-loadings and sample size.

Distribution					*N* = 300				*N* = 1,000			
	# factors	rho	Load	Cross	PA5	P10	EGA5	EGA10	PA5	PA10	EGA5	EGA10
Normal	1	0	Low	—	99	99	97	41	100	100	100	100
Normal skewed					91	100	100	26	100	100	98	100
Non-normal					100	100	100	92	100	100	100	100
Non-normal skewed					100	99	95	67	100	100	100	100
Normal	1	0	High	—	100	100	100	100	100	100	100	100
Normal-skewed					100	100	100	100	100	100	100	100
Non-normal					100	100	100	100	100	100	100	100
Non-normal skewed					100	100	100	100	100	100	100	100
Normal	2	0	Low	—	98	100	84	89	100	100	100	100
Normal-skewed					95	99	81	86	100	100	100	100
Non-normal					100	100	99	99	100	100	100	100
Non-normal skewed					97	99	97	95	100	100	100	100
Normal	2	0	High	—	100	100	100	100	100	100	100	100
Normal-skewed					100	100	100	100	100	100	100	100
Non-normal					100	100	100	100	100	100	100	100
Non-normal skewed					100	100	100	100	100	100	100	100
Normal	2	0.6	Low	Low	6	43	71	73	18	94	91	100
Normal-skewed					7	33	69	61	17	91	99	100
Non-normal					4	52	68	92	13	100	86	100
Non-normal skewed					5	33	65	74	9	98	79	100
Normal	2	0.6	High	Low	81	100	99	100	100	100	100	100
Normal-skewed					73	100	100	100	99	100	100	100
Non-normal					63	100	98	100	98	100	100	100
Non-normal skewed					59	100	97	100	96	100	100	100
Normal	2	0.6	High	High	0	3	0	71	0	21	0	88
Normal-skewed					0	27	1	72	0	73	1	86
Non-normal					0	0	0	0	0	0	0	0
Non-normal skewed					0	0	0	0	0	0	0	0
Normal	4	0	Low	—	80	98	83	91	100	100	100	100
Normal-skewed					67	98	74	88	100	100	100	100
Non-normal					95	100	96	100	100	100	100	100
Non-normal skewed					87	97	89	100	100	100	100	100
Normal	4	0	High	—	100	100	100	100	100	100	100	100
Normal-skewed					100	100	100	100	100	100	100	100
Non-normal					100	100	100	100	100	100	100	100
Non-normal skewed					100	100	100	100	100	100	100	100
Normal	4	0.6	Low	Low	0	0	13	52	0	41	5	99
Normal-skewed					0	0	16	44	0	3	31	95
Non-normal					0	0	20	87	0	2	31	100
Non-normal skewed					0	0	24	60	0	1	39	99
Normal	4	0.6	High	Low	1.5	99	100	100	63	100	100	100
Normal-skewed					0.5	94	100	100	35	98	100	100
Non-normal					0.5	55	90	100	0	100	78	100
Non-normal skewed					1	55	90	100	2	12	91	100
Normal	4	0.6	High	High	0	0	0	0	0	0	0	0
Normal-skewed					0	0	0	0	0	n/a	0	n/a
Non-normal					0	n/a	0	n/a	0	n/a	0	n/a
Non-normal skewed					0	n/a	0	n/a	0	n/a	0	n/a

Each value corresponds to the mean accuracy in factor retention after 100 reps. Distribution, Symmetrical or skewed distributions, with assumed underlying normality or non-normality; # factors, Number of latent factors (1, 2 or 4); rho, Interfactor correlations (0 or 0.6); Load, Primary loadings (low or high); Cross, Cross-loadings (low or high); PA5, Parallel Analysis for datasets with five variables per factor; PA10, Parallel Analysis for datasets with 10 variables per factor; EGA5, Exploratory Graph Analysis for datasets with five variables per factor; EGA10, Exploratory Graph Analysis for datasets with 10 variables per factor; *N*, Sample size (300 or 1,000); n/a, Impossible to generate data for this scenario.

Based on the logistic regression analysis, EGA consistently demonstrated higher predicted probabilities for accurately determining the number of factors in most scenarios examined in the study, compared to PA. Several predictors in the model were identified as highly influential, as indicated by their considerable odds ratios. Notably, the effects of some of the interactions involving each method (EGA or PA) and the levels of multiple factors were considered particularly large. These factors, ordered by effect size, included cross-loadings, primary loadings, inter-factor correlations, the number of latent factors, the number of variables per factor, and the type of generated data. Conversely, the influence of sample size and the number of categories per variable was not substantial. Due to the overwhelming number of conditions, we visualize and describe here a selection of the most informative results.

**Figure 1:**
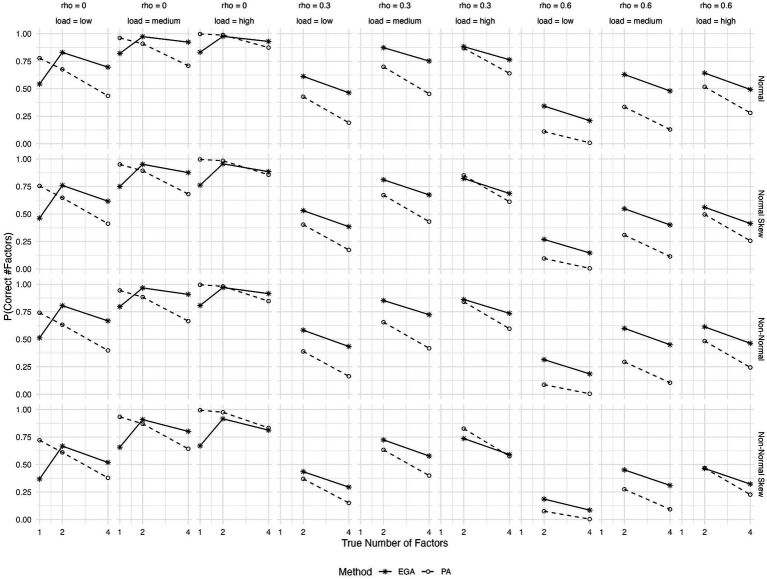
Expected probability of correctly predicting the number of factors by Exploratory Graph Analysis (EGA) and Parallel Analysis (PA) across various conditions: true number of factors (1, 2, or 4), type of generated data (symmetrical or skewed item distributions with assumed underlying normality or non-normality), level of factor loadings (low, medium or high), and level of inter-factor correlations (rho = 0, 0.3, or 0.6).

Figure 1 illustrates the expected probability of accurately predicting the number of factors using EGA and PA across different conditions: the true number of factors, the type of generated data, and the magnitudes of both primary factor loadings and inter-factor correlations. Based on the figure, as the inter-factor correlations (rho) increase, the expected probability of correctly predicting the number of factors tends to decrease for both methods. This trend is consistent and expected since higher inter-factor correlations can obscure the distinctiveness of factors, making it more challenging to identify the correct number. PA outperforms EGA in terms of predicted accuracy when the factor solution is unidimensional, regardless of the magnitude of primary loadings and the data generation process. However, its performance clearly deteriorates as the number of factors increases to 4. Moreover, in conditions where latent factors are orthogonal, that is when rho = 0, both EGA and PA demonstrate their highest probabilities of making correct predictions. However, as rho increases, EGA seems to maintain a better performance over PA. This difference between the two methods becomes more pronounced when primary factor loadings are low or medium, favoring EGA. Additionally, conditions with skewed item distributions stemming from either normal or non-normal underlying continuous variables present considerable difficulties for both methods, particularly in cases with high inter-factor correlations (rho = 0.6) and low primary loadings. However, in these scenarios EGA clearly outperforms PA.

**Figure 2:**
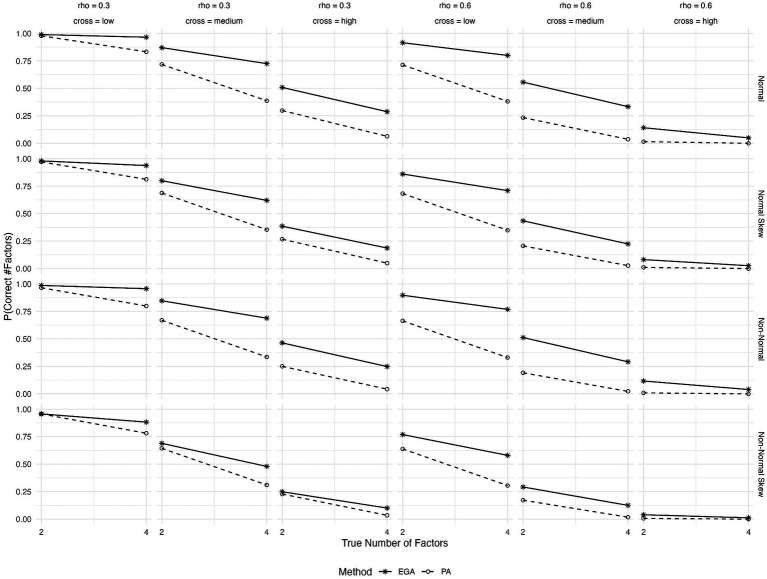
Expected probability of correctly predicting the number of factors by EGA and PA across various conditions: true number of factors (2 or 4), type of generated data (symmetrical or skewed item distributions with assumed underlying normality or non-normality), level of cross-loadings (low, medium, or high), and level of inter-factor correlations (rho = 0, 0.3, or 0.6).

The expected probabilities of correctly predicting the number of factors for different patterns of cross-loadings, inter-factor correlations, true number of factors and type of generated data are shown in Figure 2. Both methods show a decrease in the probability of correctly predicting the number of factors as cross-loadings increase from low to high. However, EGA’s decline is less steep compared to PA. This suggests that EGA may be more robust to the presence of cross-loadings, maintaining higher accuracy despite these complexities in the factor structure. However, in scenarios characterized by high cross-loadings, the efficacy of both methods markedly deteriorates. Last, the effect of item skewness or underlying non-normality on the performance of both methods is more pronounced at high inter-factor correlations (rho = 0.6) and medium or high cross-loadings.

**Figure 3:**
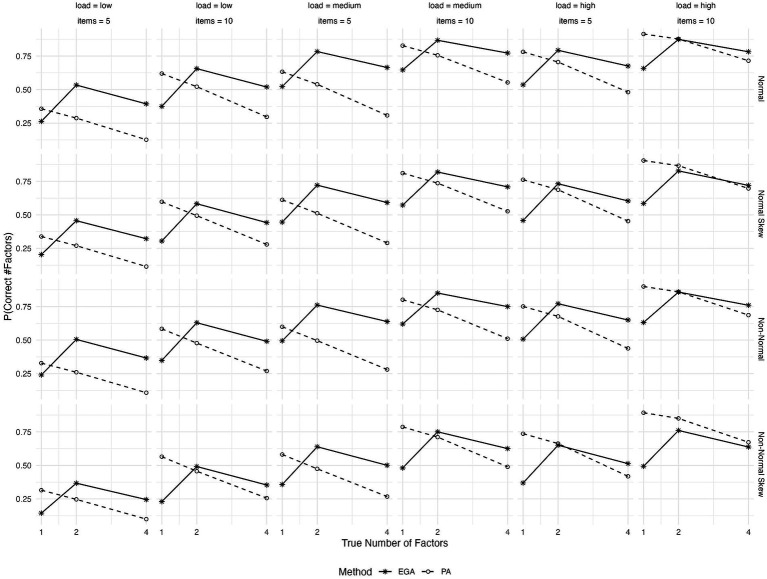
Expected probability of correctly predicting the number of factors by EGA and PA across various conditions: true number of factors (1, 2, or 4), type of generated data (symmetrical or skewed item distributions with assumed underlying normality or non-normality), level of factor loadings (low, medium, or high), and variables per factor (5 or 10).

As shown in Figure 3, the performance of both EGA and PA improves when the number of variables per factor rises from 5 to 10. PA closely matches EGA’s performance when there are 10 items per factor and high primary loadings.

## 5 Discussion

The paper evaluates the effectiveness of EGA and PA in assessing scale dimensionality in social sciences, with a focus on ordinal data. This study aims to demonstrate how these methods perform in real-world settings, where assumptions of normality may not hold. These findings are crucial for psychology and education researchers using these tools for scale development and validation, ensuring accurate representation of theoretical constructs.

Based on the simulation results, the choice between EGA and PA should be informed by the specific characteristics of the measurement scale under investigation. The performance of PA in identifying the correct number of factors significantly declined in scenarios with more than a single factor, high inter-factor correlations, moderate to high cross-loadings, or when the primary loadings were not substantial. This finding is not surprising because as factors become more correlated, the magnitude of the first eigenvalue will increase, while the remaining eigenvalues will decrease, resulting in the extraction of fewer factors (Cho et al., 2009).

On the contrary, EGA consistently demonstrated higher predicted accuracy than PA when the factor structure was less clear. This result is consistent with earlier simulation studies that contrasted EGA and PA in the context of both continuous and binary data (Golino and Epskamp, 2017; Cosemans et al., 2022). Item skewness and underlying non-normality were found to noticeably impact the performance of both methods, particularly in data with more complex factor structure.

Overall, for measurement scales that are simpler in nature, characterized by unidimensionality, minimal cross-loadings, higher factor loadings, and numerous items per factor, PA is the preferred method. For scales with more complex factor structures, however, EGA proves to be a more appropriate choice. Additionally, EGA demonstrates less variability in its performance across various levels of inter-factor correlations and cross-loadings, indicating greater consistency. This can be especially beneficial in exploratory studies where the factor structure is not well known.

In this study, while we meticulously analyzed the performance of PA and EGA using various simulated conditions, certain methodological limitations are noteworthy. The nature of the generated data, particularly our assumptions about normality and skewness, might also limit the applicability of our findings to real-world datasets, as simulated conditions cannot fully capture the complexity of actual data. Furthermore, the scope of our findings is primarily confined to the scenarios we analyzed, which may not encompass all possible variations encountered in practical applications. As such, our conclusions should be considered within the context of these specific conditions and factors. Future research directions should focus on extending this work by testing these methods under a broader range of conditions, such as scenarios with model misspecification and real-world data, to further validate and refine our understanding of PA and EGA’s performance. This approach would not only help in addressing the current study’s limitations but also contribute to the robustness and applicability of these methods in diverse research settings.

## Data availability statement

The datasets presented in this study can be found in online repositories. The names of the repository/repositories and accession number(s) can be found at: https://osf.io/hb8tw/.

## Author contributions

AM: Writing – original draft, Writing – review & editing. NT: Writing – original draft, Writing – review & editing.

## Footnotes

^1^ https://osf.io/hb8tw
