# Supplementary material for: Dimensionality assessment in ordinal data: a comparison between parallel analysis and exploratory graph analysis
Source: Front Psychol. 2024 May 6;15:1359111. doi: 10.3389/fpsyg.2024.1359111 (PMC11102999; doi:10.3389/fpsyg.2024.1359111)
Supplement: Supplementary file 1 [file Data_Sheet_1.docx]

Table S1. Logistic Regression Model Coefficients

| Variable | Estimate | Std. Error | z value | *p* |
| --- | --- | --- | --- | --- |
| Intercept | -3.4856 | 0.0666 | -52.363 | < 0.001 |
| MethodPA | 6.0975 | 0.5829 | 10.46 | < 0.001 |
| N600 | 0.4859 | 0.0205 | 23.724 | < 0.001 |
| N1000 | 0.6126 | 0.0206 | 29.719 | < 0.001 |
| rho0.3 | -3.2175 | 0.0262 | -122.7 | < 0.001 |
| rho0.6 | -7.1472 | 0.0388 | -184.32 | < 0.001 |
| distrNon-Normal Skew | -0.0088 | 0.0245 | -0.36 | 0.719 |
| distrNormal | 0.7994 | 0.0243 | 32.844 | < 0.001 |
| distrNormal Skew | 0.6427 | 0.0242 | 26.611 | < 0.001 |
| vpf10 | 3.2369 | 0.0223 | 145.283 | < 0.001 |
| pflow | -4.9688 | 0.0304 | -163.362 | < 0.001 |
| pfmedium | -1.5959 | 0.0237 | -67.462 | < 0.001 |
| sflow | 9.2959 | 0.0479 | 194.006 | < 0.001 |
| sfmedium | 4.3038 | 0.0267 | 160.917 | < 0.001 |
| cat5 | 0.0272 | 0.0167 | 1.626 | 0.104 |
| k2 | 5.8752 | 0.0678 | 86.665 | < 0.001 |
| k4 | 3.8663 | 0.0622 | 62.197 | < 0.001 |
| MethodPA:N600 | 0.8068 | 0.0301 | 26.829 | < 0.001 |
| MethodPA:N1000 | 1.4567 | 0.0312 | 46.75 | < 0.001 |
| MethodPA:rho0.3 | -0.7122 | 0.0387 | -18.385 | < 0.001 |
| MethodPA:rho0.6 | -2.4357 | 0.065 | -37.478 | < 0.001 |
| MethodPA:distrNon-Normal Skew | -0.2988 | 0.0351 | -8.511 | < 0.001 |
| MethodPA:distrNormal | -0.1924 | 0.0349 | -5.515 | < 0.001 |
| MethodPA:distrNormal Skew | -0.4746 | 0.0346 | -13.724 | < 0.001 |
| MethodPA:vpf10 | 0.7057 | 0.0342 | 20.608 | < 0.001 |
| MethodPA:pflow | -2.2328 | 0.0521 | -42.819 | < 0.001 |
| MethodPA:pfmedium | -1.3711 | 0.0352 | -38.924 | < 0.001 |
| MethodPA:sflow | 1.1690 | 0.0735 | 15.906 | < 0.001 |
| MethodPA:sfmedium | 0.5501 | 0.0415 | 13.262 | < 0.001 |
| MethodPA:cat5 | 0.0043 | 0.0242 | 0.176 | 0.860 |
| MethodPA:k2 | -7.2711 | 0.5829 | -12.473 | < 0.001 |
| MethodPA:k4 | -9.0583 | 0.5818 | -15.57 | < 0.001 |

*Note.* PA = Parallel Analysis (EGA is the reference category), pf = primary loadings (‘high’ is the reference category), rho = factor intercorrelations (0 is the reference category), sf = cross-loadings (‘high’ is the reference category), cat = number of variable categories (4 is the reference category), k = number of latent factors (1 is the reference category), vpf = variables per factor (5 is the reference category), N = sample size (300 is the reference category), distrNormal = ordinal variables generated with symmetric thresholds and assumed underlying normality, distrNormalskew = ordinal variables generated with non-symmetric thresholds and assumed underlying normality, distrNon-Normal = ordinal variables generated with symmetric thresholds and assumed underlying non-normality, (reference category), distrNon-Normal Skew = ordinal variables generated with non-symmetric thresholds and assumed underlying non-normality.

Table S2. Odds Ratios for Predictors in Logistic Regression Analysis of Factor Retention Methods

| Variable | Odds Ratio |
| --- | --- |
| sflow | 10893.48 |
| MethodPA | 444.75 |
| k2 | 356.11 |
| sfmedium | 73.98 |
| k4 | 47.76 |
| vpf10 | 25.45 |
| MethodPA:N1000 | 4.29 |
| MethodPA:sflow | 3.22 |
| MethodPA:N600 | 2.24 |
| distrNormal | 2.22 |
| MethodPA:vpf10 | 2.03 |
| distrNormal Skew | 1.9 |
| N1000 | 1.85 |
| MethodPA:sfmedium | 1.73 |
| N600 | 1.63 |
| cat5 | 1.03 |
| MethodPA:cat5 | 1 |
| distrNon-Normal Skew | 0.99 |
| MethodPA:distrNormal | 0.82 |
| MethodPA:distrNon-Normal Skew | 0.74 |
| MethodPA:distrNormal Skew | 0.62 |
| MethodPA:rho0.3 | 0.49 |
| MethodPA:pfmedium | 0.25 |
| pfmedium | 0.2 |
| MethodPA:pflow | 0.11 |
| MethodPA:rho0.6 | 0.09 |
| rho0.3 | 0.04 |
| (Intercept) | 0.03 |
| pflow | 0.01 |
| rho0.6 | 0 |
| MethodPA:k2 | 0 |
| MethodPA:k4 | 0 |

*Note.* PA = Parallel Analysis (EGA is the reference category), pf = primary loadings (‘high’ is the reference category), rho = factor intercorrelations (0 is the reference category), sf = cross-loadings (‘high’ is the reference category), cat = number of variable categories (4 is the reference category), k = number of latent factors (1 is the reference category), vpf = variables per factor (5 is the reference category), N = sample size (300 is the reference category), distrNormal = ordinal variables generated with symmetric thresholds and assumed underlying normality, distrNormalskew = ordinal variables generated with non-symmetric thresholds and assumed underlying normality, distrNon-Normal = ordinal variables generated with symmetric thresholds and assumed underlying non-normality, (reference category), distrNon-Normal Skew = ordinal variables generated with non-symmetric thresholds and assumed underlying non-normality.
